# Factors Associated With Health Service Utilization in Ulaanbaatar, Mongolia: A Population-Based Survey

**DOI:** 10.2188/jea.JE20120123

**Published:** 2013-09-05

**Authors:** Amarsanaa Gan-Yadam, Ryoji Shinohara, Yuka Sugisawa, Emiko Tanaka, Taeko Watanabe, Maki Hirano, Etsuko Tomisaki, Kentaro Morita, Yoko Onda, Kentaro Tokutake, Yukiko Mochizuki, Misako Matsumoto, Chihiro Sugita, Tokie Anme

**Affiliations:** 1 International Community Care and Life Span Development: Empowerment Sciences, Graduate School of Comprehensive Human Sciences, University of Tsukuba, Tsukuba, Ibaraki, Japan; 2 Research Fellow, Japan Society for the Promotion of Science, Tokyo, Japan; 3 Philanthropy Centre for Children and the Elderly, Ulaanbaatar, Mongolia; 4 Department of Health Sciences, Interdisciplinary Graduate School of Medicine and Engineering, University of Yamanashi, Chuo, Yamanashi, Japan; 5 Ushiku Health Centre, Ushiku, Ibaraki, Japan

**Keywords:** health service utilization, equity, Mongolia

## Abstract

**Background:**

Understanding patterns of health service utilization can improve health care and increase use of health services. We examined patterns of health service utilization among residents of Ulaanbaatar, Mongolia.

**Methods:**

A total of 500 adults were surveyed using paper-based questionnaires. The χ^2^ test and multiple logistic regression were used to identify associations between factors.

**Results:**

44.1% of respondents had visited a physician during the previous 12 months. After controlling for determinants, the significant predictors of utilization of health service were attention to health examinations (OR = 3.6, CI: 1.93–6.76), being married (OR = 2.7, CI: 1.50–4.72), being satisfied with the overall cleanliness of the hospital (OR = 2.4, CI: 1.12–5.19), being a nonsmoker (OR = 2.2, CI: 1.21–3.98), having periodic physical examinations (OR = 2.2, CI: 1.25–3.71), not being a hospital patient during the previous 3 years (OR = 2.1, CI: 1.22–3.73), having proper documentation (OR = 1.9, CI: 1.10–3.43), having medical insurance (OR = 1.9, CI: 1.96–3.28), not wanting to receive information on food and nutrition (OR = 0.6, CI: 0.36–0.96), having more than 5 household members (OR = 0.5, CI: 0.50–0.85), low income (OR = 0.5, CI: 0.30–0.85), lack of concern for food and nutrition (OR = 0.5, CI: 0.28–0.84), self-medication during the past 12 months (OR = 0.4, CI: 0.24–0.69), and desire for treatment abroad (OR = 0.4, CI: 0.20–0.60).

**Conclusions:**

A number of health-related behaviors and sociodemographic factors were important predictors of health service utilization.

## INTRODUCTION

Health status and health service utilization vary according to social, economic, cultural, demographic, and geographic conditions.^1^ Many countries seek to increase utilization of health services and promote equitable access to health care, especially in the developing world.^2^^,^^3^ To increase health service utilization and improve health status, one needs to understand the forces that encourage and inhibit health service utilization. Policymakers need to identify patterns of health-seeking behaviors and health service utilization, to ensure fair access to health care services.^4^

In the developed world, several studies evaluated health service utilization.^5^^,^^6^ Many studies have attempted to identify important factors and design the best models to identify key variables in connection with health service utilization.^7^^–^^9^ A variety of factors have been identified as determinants of utilization of health services, including socio-demographic status, cultural beliefs, economic conditions, health service satisfaction, health status, and health service issues.^2^^,^^10^^,^^11^ According to Andersen’s model of behavioral health service, utilization of health services involves 3 components: primary determinants, health behavior, and health outcome factors.^7^^,^^8^ Primary determinants include socio-demographic information, the health care system, and political, physical, and economic influences. Health behavior factors include personal health behaviors, lifestyle factors, social motivation, and use of health services. Self-assessed health, health service satisfaction, and evaluated health status are health outcome factors.

Several studies have shown that low socio-economic status, older age, gender disparities, low education level, large family size, and limited physical and financial accessibility result in poor health service utilization. A study in the former Soviet Union found that lack of money was the most important reason for not seeking care.^1^ Numerous studies reported that poor health status, type of illness, and poor self-assessed health influenced utilization of health services.^12^^,^^13^ Furthermore, some research has shown that dissatisfaction with health services leads to less utilization of health services.^14^ In many developing countries, physical accessibility, infrastructure (including hospital location), and availability of transportation influence health service utilization.^2^^,^^15^ A smaller number of studies found that communication barriers, such as linguistic and cultural gaps, led to poor health utilization, self-medication, self-care, and home-based treatment.^16^

### Health status and the Mongolian health care system

Mongolia is located in Central Asia and borders China and the Russian Federation. The total population in 2010 was 2.75 million people, and the population is spread thinly over a territory of 1 566 500 square kilometers.^17^

Mongolia is a post-communist state and had a socialist health care system until the collapse of the socialist regime, in 1990. Since its transition to democracy and a market economy, Mongolia has undergone a series of health care reforms. As a result of comprehensive government policies and programs, administered by its agencies and institutes, major health indicators have improved.^18^ Although the government is the main provider of modern health services, the private sector also provides a moderate level of modern health services. In addition to the modern health care delivery system, traditional treatments such as bone setters, herbal remedies, and Buddhist and Shaman rituals still exist.

At the end of 2010, the nationwide medical service consisted of 16 specialized hospitals, 4 regional diagnosis and treatment centers, 17 provincial general hospitals, 12 district general hospitals, 6 rural general hospitals, 37 inter-town hospitals, 274 town hospitals, 218 family group practices, and 1113 private clinics.^19^ Regarding human health resources, there were 2.7 physicians, 3.3 nurses, and 0.4 pharmacists per 1000 population.^19^ While the number of human health resources is quite high as a proportion of the Mongolian population, there are few health professionals in rural and semirural areas. In addition to the health centers, nongovernmental organizations (NGOs) have been active for many years in Mongolia, implementing various health-related projects, including programs in nutrition, child care and maternal health, immunization, prevention, behavior change, poverty reduction, and capacity development. The national statistical survey of 2010 reported that diseases of the circulatory system, neoplasms, diseases of the respiratory system, and injury/poisoning were the leading causes of morbidity and mortality.^20^ Smoking, alcohol consumption, unbalanced diet, and physical inactivity were reported to be the major risk factors for morbidity and mortality and remain prevalent among the population.^19^

To strengthen the health system, improve community health status, and promote equitable access to health care service, it is essential to understand the factors associated with health service utilization. We investigated the patterns of health service utilization among local residents of Ulaanbaatar, the capital city of Mongolia.

## METHODS

### Study participants and sampling

A community based cross-sectional study was conducted among urban and suburban residents of Ulaanbaatar, Mongolia. According to 2012 statistics, there were 1 206 610 residents, 45.8% of the residential population of the country, living in the city; 571 192 were male and 635 418 were female. Approximately 67.2% of the total population of the city was aged 16 to 59 years, and only 6.2% were aged 60 years or older. Regarding residence location, 60% of the population of Ulaanbaatar live in the *ger* districts (suburban areas); the remaining 40% live in residential areas and are housed in apartment blocks connected to a centralized substructure. As of 2012, 40.4% of all Mongolian families live in Ulaanbaatar, and the average number of persons per family is 3.9.^21^ A household socioeconomic survey in 2011 showed that around 23.5% of the population of the city live below the poverty line and that the unemployment rate of the country is 15.3%.^22^ Among the population aged 15 years or older, 31.3% are single (never married), 60.1% are married, and 8.6% are divorced or widowed. Among the population aged 10 years or older, 92.5% have at least a primary education (4 years of education).^17^

A multistage sampling technique was used to represent the city population. First, 3 districts were selected (Songinokhairkhan, Khan-Uul, and Nalaikh) by lottery. Then, on the basis of geographic area and probability proportion, 9 subdistricts (3 from each of the selected districts) were included in the study. Next, 9 *baghs* (the smallest administrative division) were randomly selected. Finally, using the list of households from the selected *baghs*, 500 households were selected by lottery to create the final sample. Sampled households were visited by trained social work students, and Kish tables (which ensure equal probability) were used to select respondents within the household. The data collectors were responsible for selecting respondents and addressing misunderstandings. A pre-tested, self-administered, paper-based questionnaire was given to individuals aged 18 or older, as they were judged to be old enough to make their own decisions concerning health care. Questionnaires with missing data for any item were excluded. Ultimately, the final sample size was 465 respondents.

This study was approved by the Ethics Committee of the Graduate School of Comprehensive Human Sciences, University of Tsukuba.

### Variables and analytic strategy

A visit to a physician during the past 12 months was used as an index of health service utilization. Andersen’s model^7^ of health utilization was used to predict health service utilization. We applied the model to the local community in Mongolia, after considering local context and cultural sensitivities.

The cut-points were determined before testing the statistics. For example, age was divided into 2 groups (18–59 vs ≥60 years) because of the age range, life expectancy rate, and culture of the participants. Mongolia has a young population, with a median age of 25.4 years.^23^ Life expectancy at birth is 68 years for both sexes,^20^ and 60 is considered old age among Mongolians. Participant income was also classified into 2 groups with reference to the minimum subsistence level in Mongolia.^24^ Participants with an income lower than the minimum subsistence level (approximately 90 USD per month) were defined as poor in this study.

All statistical analyses were performed using the Statistical Analysis System (SAS 9.1). The χ^2^ test was used to test for associations between variables. Factors found to be statistically significant on the χ^2^ test were analyzed by multiple logistic regression analysis to identify significant predictors of health care utilization. Adjusted and unadjusted odds ratios (ORs) with 95% CIs were reported, and adjusted ORs were computed using variables that were statistically significant on the χ^2^ test. Associations were evaluated using a significance level of p less than 0.05. Tests of interactions and collinearity (variance inflation factor <10) were also used.

## RESULTS

The study respondents were 465 adults: 185 men (39.8%) and 280 women (60.2%). The respondents ranged in age from 18 to 83 years, and mean age was 37.0 years. Approximately 44.1% of respondents had visited a physician for a general health checkup during the past 12 months. The χ^2^ test showed significant associations between health care utilization and all primary determinants except employment status, education, and residence location (Table 1). Men (*P* = 0.027), younger people (*P* = 0.005), unmarried people (*P* = 0.001), and individuals who were not poor (*P* = 0.034) were less likely to use health services. In contrast, individuals from families with more than 5 people (*P* = 0.010) and those who lived in 1 place for longer than 4 years were more likely to use health services. Table 2 shows the relationship between health behavior factors and health service utilization. Use of hospital services was lower among smokers (*P* = 0.009) and people who were unconcerned by their diet (*P* = 0.050) and weight (*P* = 0.044), as compared with nonsmokers and people who were concerned with their diet and weight for health reasons. However, individuals who paid attention to health examinations were more likely to use health services than those who were not (*P* = 0.001). In addition, health services were more often utilized by respondents who had periodic dental (*P* = 0.023) and physical examinations (*P* < 0.001). Moreover, there was a statistically significant positive association with use of media such as internet (*P* = 0.033) and radio (*P* = 0.021). However, respondents who desired information on food and nutrition (*P* = 0.053) or child health care (*P* = 0.041), were much less likely to have visited a physician than those who did not seek such information. Regarding social motivation, respondents who participated in group support activities were more likely to visit a physician (*P* = 0.015). In addition, those who volunteered to help others to improve local problems or health status during the previous 12 months were more likely to visit a physician (*P* = 0.004).

**Table 1.** Primary determinants of health service utilization

Items	*n*	Visited physician during past 12 months?		*P*

		Yes	No	
Marital status				
Married^a^	319 (68.6)	159 (49.8)	160 (50.2)	0.001
Unmarried	146 (31.4)	46 (31.5)	100 (68.5)	
Age, years				
18–59	425 (91.4)	179 (42.1)	246 (57.9)	0.005
≥60	40 (8.6)	26 (65.0)	14 (35.0)	
Household size				
≤4	277 (59.6)	107 (38.6)	170 (61.4)	0.01
≥5	188 (40.4)	98 (52.1)	90 (47.9)	
Sex				
Male	185 (39.8)	70 (37.8)	115 (62.2)	0.027
Female	280 (60.2)	135 (48.2)	145 (51.8)	
Self-reported income level				
Not poor	170 (36.6)	64 (37.6)	106 (62.4)	0.034
Poor	295 (63.4)	141 (47.8)	154 (52.2)	
Duration of residence in 1 place, years				
≤3	142 (30.5)	53 (37.3)	89 (62.7)	0.052
≥4	323 (69.5)	152 (47.1)	171 (52.9)	
Employment status				
Employed^b^	374 (80.4)	159 (42.5)	215 (57.5)	0.166
Unemployed	91 (19.6)	46 (50.5)	45 (49.5)	
Residence location				
Downtown	174 (37.4)	79 (45.4)	95 (54.6)	0.658
Suburban	291 (62.6)	126 (43.3)	165 (56.7)	
Education				
High^c^	410 (88.2)	180 (43.9)	230 (56.1)	0.828
Low	55 (11.8)	25 (45.5)	30 (54.5)	

Values represent number (%).

^a^Includes both divorced and widowed adults.

^b^Includes both students and pensioners.

^c^Denotes ≥10 years of education.

**Table 2.** Health behavior factors associated with health service utilization

Items	*n*	Visited physician during past 12 months?		*P*

		Yes	No	
Self-medication during past 12 months				
Yes	242 (52.0)	71 (29.3)	171 (70.7)	<0.001
No	223 (48.0)	134 (60.1)	89 (39.9)	
Have periodic physical examinations				
Yes	255 (54.8)	145 (56.9)	110 (43.1)	<0.001
No	210 (45.2)	60 (28.6)	150 (71.4)	
Been a patient in hospital in previous 3 years (respondent or family member)				
Yes	275 (59.1)	146 (53.1)	129 (46.9)	<0.001
No	190 (40.9)	59 (31.1)	131 (68.9)	
Have medical insurance				
Yes	288 (61.9)	145 (50.4)	143 (49.6)	0.001
No	177 (38.1)	60 (33.9)	117 (66.1)	
Pay attention to health examinations				
Yes	88 (18.9)	55 (62.5)	33 (37.5)	0.001
No	377 (81.1)	150 (39.8)	227 (60.2)	
Volunteered to help others during past 12 months				
Yes	45 (9.7)	29 (64.4)	16 (35.6)	0.004
No	420 (90.3)	176 (41.9)	244 (58.1)	
Smoking habit				
Smoker	138 (29.7)	48 (34.8)	90 (65.2)	0.009
Nonsmoker	327 (70.3)	157 (48.0)	170 (52.0)	
Visited friend or loved one in hospital during past 12 months				
Yes	257 (55.3)	127 (49.4)	130 (50.6)	0.01
No	208 (44.7)	78 (37.5)	130 (62.5)	
Want to participate in group support activities				
Yes	44 (9.5)	27 (61.4)	17 (38.6)	0.015
No	421 (90.5)	178 (42.3)	243 (57.7)	
Radio use				
Yes	239 (51.4)	93 (38.9)	146 (61.1)	0.021
No	226 (48.6)	112 (49.6)	114 (50.4)	
Lack of legal documents as reason for not visiting health facility				
Yes	122 (26.2)	43 (35.3)	79 (64.7)	0.022
No	343 (73.8)	162 (47.2)	181 (52.8)	
Have periodic dental examination				
Yes	256 (55.0)	125 (48.8)	131 (51.2)	0.023
No	209 (45.0)	80 (38.3)	129 (61.7)	
Internet use				
Yes	221 (47.5)	86 (38.9)	135 (61.1)	0.033
No	244 (52.5)	119 (48.8)	125 (51.2)	
Get health-related instruction from religious people				
Yes	144 (31.0)	74 (51.4)	70 (48.6)	0.034
No	321 (69.0)	131 (40.8)	190 (59.2)	
Want information on child health care				
Yes	44 (9.5)	13 (29.5)	31 (70.5)	0.041
No	421 (90.5)	192 (45.6)	229 (54.4)	
Pay attention to weight				
Yes	62 (13.3)	20 (32.3)	42 (67.7)	0.044
No	403 (86.7)	185 (45.9)	218 (54.1)	
Pay attention to food and nutrition				
Yes	343 (73.8)	142 (41.4)	201 (58.6)	0.05
No	122 (26.2)	63 (51.6)	59 (48.4)	
Want information on food and nutrition				
Yes	191 (41.1)	74 (38.7)	117 (61.3)	0.053
No	274 (58.9)	131 (47.8)	143 (52.2)	

Values represent number (%).

Respondents were also asked to give reasons for not seeking health services, and 26.2% reported that lacking proper documentation was a reason for not using health services (*P* = 0.022). Furthermore, health-seeking behavior was related to lack of medical insurance (*P* = 0.001), receiving health instruction from religious people (*P* = 0.034), and self-medication (*P* < 0.001). Interestingly, respondents who had visited a friend or loved one in hospital during the previous 12 months (*P* = 0.010) and those who had been hospitalized or had a family member in hospital during the previous 3 years were more likely to visit physicians (*P* < 0.001).

With regard to health outcome factors, the reasons for seeking health services were related to health service satisfaction, physician skills, trust in the local hospital service, and self-assessed health status (Table 3). Respondents with poor self-assessed health status (*P* = 0.002) and those with self-assessed long-standing illness (*P* = 0.001) were more likely to visit physicians. Respondents who had been hospitalized were asked to provide additional data, and there was a significant association with physician abilities and skills (*P* = 0.006). In addition, many respondents agreed that treatment abroad was better than treatment in Mongolia (*P* = 0.011). Satisfaction with hospital service was very low among respondents, and those who were satisfied with health services were more likely to visit a physician and use such services.

**Table 3.** Health outcome factors associated with health service utilization

Items	*n*	Visited physician during past 12 months?		*P*

		Yes	No	
Satisfied with hospital equipment				
Yes	72 (15.5)	47 (65.3)	25 (34.7)	<0.001
No	393 (84.5)	158 (40.2)	235 (59.8)	
Satisfied with overall cleanliness of hospital				
Yes	63 (13.6)	41 (65.1)	22 (34.9)	0.001
No	402 (86.4)	164 (40.8)	238 (59.2)	
Satisfied with skills of hospital staff				
Yes	173 (37.2)	92 (53.2)	81 (46.8)	0.002
No	292 (62.8)	113 (38.7)	179 (61.3)	
Satisfied with hospital room facilities				
Yes	82 (17.6)	49 (59.8)	33 (40.2)	0.002
No	383 (82.4)	156 (40.7)	227 (59.3)	
Satisfied with hospital location				
Yes	182 (39.1)	95 (52.2)	87 (47.8)	0.005
No	283 (60.9)	110 (38.9)	173 (61.1)	
Self-assessed long-standing illness				
Yes	228 (49.0)	83 (36.4)	145 (63.6)	0.001
No	237 (51.0)	122 (51.5)	115 (48.5)	
Self-assessed health				
Good	249 (53.6)	93 (37.4)	156 (62.6)	0.002
Poor	216 (46.4)	112 (51.8)	104 (48.2)	
Categories for hospitalization (physician ability and skills)				
Yes	412 (88.6)	191 (46.4)	221 (53.6)	0.006
No	53 (11.4)	14 (26.4)	39 (73.6)	
Desire to be treated abroad				
No	187 (40.2)	69 (36.9)	118 (63.1)	0.011
Yes	278 (59.8)	136 (48.9)	142 (51.1)	

Values represent number (%).

Logistic regression analysis showed (Table 4) that married people (OR = 2.66, CI: 1.50–4.72), those with a household size greater than 5 (OR = 0.53, CI: 0.50–0.85), and those with a low income (OR = 0.50, CI: 0.30–0.85) were more likely to visit a physician. Regarding health behavior, nonsmokers were 2.19 times as likely as smokers to use health services (CI: 1.21–3.98). Furthermore, those who were unconcerned about food and nutrition were less likely to visit a physician (OR = 0.48, CI: 0.28–0.84). Respondents who sought health examinations were 3.58 times as likely to have visited a physician (CI: 1.93–6.76). In addition, people who do not seek information on food and nutrition were 0.59 times as likely to use health services (CI: 0.36–0.96) as compared with those who sought such information. Health service use was also related to medication use, and people who self-medicated were 0.41 times as likely to have visited physicians than those who had not (CI: 0.24–0.69). People with medical insurance (OR = 1.9, CI: 1.96–3.28), those who sought periodic physical exams (OR = 2.2, CI: 1.25–3.71), and those who had not been hospitalized during the previous 3 years (OR = 2.1, CI: 1.22–3.73) were more likely to use health services. Additionally, respondents with proper documentation were 1.94 times as likely to use health services as those without such documentation (CI: 1.10–3.43). After adjustment for health outcome factors, only 2 variables were independently associated with utilization of health services: individuals who were satisfied with the overall cleanliness of the hospital were 2.40 times as likely as those who were not to use health services (CI: 1.12–5.19), and people who did not trust domestic health services were 0.35 times as likely to use health services as those who did (OR = 0.4, CI: 0.20–0.60).

**Table 4.** Multiple logistic regression analysis of factors associated with health service utilization

Variables	Visited physician during past 12 months?	

	Unadjusted (95% CI)	Adjusted^a^ (95% CI)
**Primary determinants**		
Marital status (married)	2.16 (1.43–3.26)	2.66 (1.50–4.72)
Sex (female)	0.65 (0.45–0.96)	1.13 (0.66–1.94)
Age (≥60 years)	0.39 (0.20–0.77)	0.86 (0.34–2.19)
Household size (>5 members)	0.58 (0.40–0.84)	0.53 (0.50–0.85)
Self-reported low income	0.66 (0.45–0.97)	0.50 (0.30–0.85)
Duration of residence in 1 place (lived in 1 place longer than 5 years)	1.49 (1.00–2.24)	1.10 (0.64–1.89)
**Health behavior**		
Paying attention to health examination	2.52 (1.56–4.07)	3.61 (1.93–6.76)
Nonsmoker	1.73 (1.15–2.61)	2.20 (1.21–3.98)
Having periodic physical examination	3.30 (2.23–4.86)	2.15 (1.25–3.71)
Not being a patient in a hospital during past 3 years (family member or respondent)	2.51 (1.71–3.70)	2.13 (1.22–3.73)
Having medical insurance	1.98 (1.34–2.91)	1.96 (1.17–3.28)
Having legal documentation is reason for visiting health facility	1.64 (1.07–2.52)	1.95 (1.10–3.43)
Self-medication during past 12 months	0.28 (0.19–0.41)	0.41 (0.24–0.69)
Not concerned with food and nutrition	0.66 (0.44–1.00)	0.48 (0.28–0.84)
No desire for information on food and nutrition	0.69 (0.47–1.01)	0.59 (0.36–0.96)
No desire for information on child health care	0.50 (0.26–0.98)	0.58 (0.24–1.36)
Desire to participate in group support activities	2.17 (1.15–4.10)	1.52 (0.66–3.49)
Not using internet	0.67 (0.46–0.97)	1.18 (0.66–2.09)
Not using radio	0.65 (0.45–0.94)	0.85 (0.51–1.41)
Not paying attention to weight	0.56 (0.32–0.99)	0.54 (0.26–1.12)
Having periodic dental examinations	1.54 (1.06–2.23)	1.15 (0.67–1.98)
Volunteered to help others during past 12 months	2.51 (1.32–4.77)	1.80 (0.79–4.12)
Visited friend or loved one in hospital during past 12 months	1.63 (1.12–2.36)	0.82 (0.48–1.42)
Received health-related instruction from religious people	0.65 (0.44–0.97)	1.18 (0.69–1.99)
**Health outcomes**		
Desire to be treated abroad	0.61 (0.42–0.89)	0.35 (0.20–0.60)
Satisfied with overall cleanliness of hospital	2.71 (1.55–4.71)	2.41 (1.12–5.19)
Satisfied with hospital equipment	2.80 (1.65–4.73)	1.75 (0.83–3.69)
Satisfied with hospital staff skills	1.80 (1.23–2.63)	1.19 (0.69–2.05)
Satisfied with hospital location	1.72 (1.18–2.50)	1.17 (0.70–1.98)
Satisfied with hospital room facilities	2.16 (1.33–3.51)	0.96 (0.47–1.99)
Self-assessed poor health	0.55 (0.38–0.80)	1.22 (0.68–2.19)
Self-assessed long-standing illness	0.54 (0.37–0.78)	0.80 (0.45–1.42)
Categories for hospitalization (not concerned with physician ability and skills)	0.42 (0.22–0.79)	0.64 (0.28–1.43)

Values represent odds ratios and 95% CI.

^a^Odds ratios adjusted for all variables in table.

## DISCUSSION

We analyzed patterns of health service utilization among Mongolian adults and found that 44% of respondents had used health services during the previous 12 months. This figure is lower than in other post-socialist countries^1^ and indicates that utilization must improve in urban and suburban Mongolia. Greater utilization of health services was observed among married people. Also, people from larger families were more likely to seek health care. The finding among married people may be due to the fact that most unmarried respondents in this study were young and in better health than married respondents. In addition, family ties and responsibilities, including caring for elders and relatives, are important in Mongolia, and the nomadic origin of Mongolian culture may affect social interaction, including health utilization, in married and larger families.

Higher income was associated with reduced use of health services in this study; however, many previous studies found that poor people utilized such services less often than people with higher incomes.^25^ In Mongolia, poor people have more health problems than those with higher incomes,^26^ and 38.7% of the Mongolian general population was living below the national poverty line in 2009.^27^ A 2007 survey by the Asian Development Bank revealed that 58% of all clients of family group practices (FGPs) were poor people and that nearly 70% of FGP workloads were taken up by children and elderly adults.^28^ In addition, primary health service in Mongolia is free for socially vulnerable groups, which include elderly adults, single parents, children younger than 16 years, and unemployed people.

While poor people are the majority of FGP clients, they may not receive secondary and tertiary health services because they often lack health insurance and proper documentation, thus limiting access to health services. This study revealed that having health insurance and proper documentation were important in health service utilization. Migrants from the countryside and poor families in suburban areas are less likely to have proper documentation.

People living in suburban areas are more likely to be poor than those living in urban areas^24^; however, there was no statistically significant relationship between health service utilization and residence location in the present study, perhaps because Ulaanbaatar is smaller than other capital cities; thus, the distance to the nearest FGP or health center is not great and physical accessibility to health services might not influence health service utilization among urban and suburban residents. In addition, education was not significantly associated with health service utilization in the present study, probably because Mongolia has a high education level and the literacy rate among Mongolians aged 15 years or older is 98.3%.^17^

Another important factor in this study was self-medication, which was associated with utilization of health services. The self-medication rate was very high in the community, and self-medication has an important role in health care and health service utilization in Mongolia. Many studies have shown that poverty leads to self-care and self-medication, which affects health service utilization.^29^^,^^30^

We also found widespread dissatisfaction with health care services among urban and suburban Mongolian communities. Other studies have noted that client satisfaction affected the decision to seek care.^31^ When satisfaction with the overall cleanliness of hospitals was prevalent, people were 2.4 times as likely to use health services in this study. Furthermore, trust in domestic health care was very low, and many people felt that treatment abroad was much better than treatment in Mongolia. Numerous patients are treated abroad each year, and the number is increasing. However, there are no clear data on treatment abroad, which thus needs to be further examined.

Regarding health behaviors, the rate of periodic health examination and attention to health also seemed to be unsatisfactory among our respondents. People who sought to maintain their health by paying attention to food and nutrition had less health service utilization. However, people who did not desire information on food and nutrition also had less health service utilization. These findings can be explained by the fact that individuals who are not careful about their diet may have poorer health status. Mongolians consume much meat, and most have a high intake of salt and low intake of fruit and vegetables.^32^ People who had periodic physical examinations and paid attention to health examinations were more likely to use health services, perhaps because they had poorer health than those who did not have periodic examinations.

In Mongolia, respondents who had been patients in a hospital were more likely to visit a physician. Hospital patients may be more prone to illness and are more likely to have ongoing relationships with physicians. Smoking was also related to health service utilization in this study: nonsmokers were more likely to visit a physician. According to the World Health Organization, the smoking rate for Mongolians aged 15 years or older was 43% among males and 5.2% among females.^33^ Smoking is a worsening problem among the Mongolian population, and incidences of tobacco-related diseases are increasing. Therefore, more efforts are needed to promote smoking prevention and cessation as an approach to improving the health status of the local community and encourage use of health services.

A number of limitations in this study should be noted. We considered health service utilization only among adult respondents. We did not control for institutional factors that might influence utilization of services. In addition, we did not control for or use hospital diagnoses, and we did not assess clinical need. Multiple visits to health facilities were not considered. In addition, we described health service utilization patterns only for this relatively small study population. Thus, the present findings cannot be generalized beyond the study groups and areas. The study was a “snapshot” survey and cannot identify trends in utilization of health care services. Therefore, we were only able to examine associations between dependent and independent variables. In addition, the study design did not allow for inference of causality. Furthermore, the data source for this study was self-reported information from the respondents. The information provided was not validated by an objective source. Recall bias is a possibility because evaluation of self-reported information and behavior patterns was retrospective. Thus, the respondents might have forgotten some of their experiences and previous visits to health facilities.

Despite these constraints, the study has provided important information on patterns of health service utilization. It is important to note that very few studies of this type have been conducted in Mongolia, so comparison with previous studies was not possible.

## CONCLUSIONS

To improve and develop local health services and health policy in Mongolia, we need to understand the community, its health seeking behaviors, and the factors that encourage and deter health service utilization. We identified predictors of health service utilization in the developing country of Mongolia and found that the rate of health service utilization was unsatisfactory. Helping local residents to improve their health-related behaviors and empowering the community may improve utilization of health services. In addition, improved trust in medical care and greater satisfaction with health services may increase use of health services. However, local health services and health promotion activities must also improve. In addition, a comprehensive health care system must focus on elderly adults, women, and poor families.

We recommend careful consideration of the patterns found to be statistically significant with regard to health service utilization in this study. It is hoped that our findings will inspire future research, have an impact on the design and implementation of health reforms, and empower the community and health care system in Mongolia.
